# Subjective Positive and Negative Sleep Variables Differentially Affect Cellular Immune Activity in a Breast Cancer Survivor: A Time-series Analysis Approach

**DOI:** 10.3389/fneur.2017.00693

**Published:** 2018-01-09

**Authors:** Magdalena Singer, Christina Burbaum, Kurt Fritzsche, Sylvia Peterlini, Harald R. Bliem, Francisco M. Ocaña-Peinado, Dietmar Fuchs, Christian Schubert

**Affiliations:** ^1^Clinical Department of Medical Psychology, Medical University of Innsbruck, Innsbruck, Austria; ^2^Department of Psychology, University Innsbruck, Innsbruck, Austria; ^3^Department of Rehabilitation Psychology and Psychotherapy, University of Freiburg, Freiburg, Germany; ^4^Department of Psychosomatic Medicine and Psychotherapy, University Hospital Freiburg, Freiburg, Germany; ^5^Department of Statistics and Operations Research, University of Granada, Granada, Spain; ^6^Division of Biological Chemistry, Biocenter, Medical University of Innsbruck, Innsbruck, Austria

**Keywords:** cancer, sleep, neopterin, time-series analysis, single-case design

## Abstract

This study on a breast cancer survivor suffering from cancer-related fatigue (CaRF) and depression investigated the bidirectional relationship between cellular immune activity and subjective sleep. The 49-year-old patient (breast cancer diagnosis 5 years before the study, currently in remission) collected her full urine output for 28 days in 12-h intervals (8:00 p.m. to 8:00 a.m. and 8:00 a.m. to 8:00 p.m.). These urine samples were used to determine urinary neopterin (cellular immune activation marker) and creatinine concentrations *via* high-pressure liquid chromatography (HPLC). Each morning, the patient answered questions on five sleep variables: sleep quality (SQ), sleep recreational value (SRV), total sleep time (TST), total wake time (TWT), and awakenings during sleep period (ADS). For the purpose of this study, the time series of the nighttime urinary neopterin levels and the five sleep variables were determined. Using centered moving average (CMA) smoothing and cross-correlational analysis, this study showed that increases in the positive sleep variables SQ and SRV were followed by urinary neopterin concentration decreases after 96–120 h (SQ, lag 4: *r* = −0.411; *p* = 0.044; SRV: lag 4: *r* = −0.472; *p* = 0.021) and 120–144 h (SRV, lag 5: *r* = −0.464; *p* = 0.026). Increases in the negative sleep variable TWT, by contrast, were followed by increases in urinary neopterin concentrations 72–96 h later (lag 3: *r* = 0.522; *p* = 0.009). No systematic effects in the other direction, i.e., from urinary neopterin levels to sleep, were observed in this study. Although preliminary, the findings of this study highlight the benefit of carefully investigating temporal delays and directions of effects when studying the dynamic relationship between sleep and immune variables in the natural context of everyday life.

## Introduction

In this study, we investigated the bidirectional relationship between cellular immune activity and subjective sleep in a breast cancer survivor suffering from cancer-related fatigue (CaRF) and depression.

The patient under study, a 49-year-old Caucasian woman with the German Abitur qualification (high school diploma with university entrance examination), is married and has three children. She has suffered chronically from dysthymia (F34.1). About 5 years before this study began, the patient was diagnosed with a ductal mamma carcinoma (C50.4) of her right breast [pT2, pN1biv (6 of 13), cM0, G3, R0, ER 10%, PR 70–80%, HER2+/neu+, score = 3]. For primary cancer therapy, the patient underwent surgery, radiotherapy and antiestrogen therapy (tamoxifen). Antiestrogen therapy ended 6 months before study start. At study start, the patient was clinically free of evidence of metastatic or recurrent lesions. Since cancer diagnosis and therapy, the patient has been suffering from severe CaRF. CaRF symptoms persisted until study start and beyond. Furthermore, chronic depressive disorder symptoms (dysthymia, F34.1) increased since cancer diagnosis and therapy, leading to an additional clinical diagnosis of adjustment disorder with depressed mood (F43.21) at the start of the study. Aside from these health issues, the patient has no history of immunological or endocrinological disease or health conditions that could affect the immune and endocrine systems.

As to the patient’s subjective assessment of her sleep before study start, the Center for Epidemiologic Studies Depression Scale (in German) revealed that her sleep was restless on 5–7 days during the preceding week (score 4 on a 4-point Likert scale). For the same week, the EORTC QLQ-C30 (version 3, in German) showed that the patient had moderate sleep difficulties (score 3 on a 4-point Likert scale).

## Background

Long-term behavioral symptoms are prominent in cancer patients (1). They can persist many years after primary treatment, entail various psychological and medical consequences, and lead to a reduction in quality of life (1, 2). They may also facilitate cancer recurrence (1, 2).

One of these behavioral symptoms is sleep disturbance, i.e., symptoms of “perceived or actual alterations in night sleep with resultant daytime impairment” (3, 4). Sleep disturbances affect about 50% of breast cancer survivors (5). In this article, the term “cancer survivor” refers to patients who have finished primary cancer treatment (6).

Emerging evidence suggests that cancer-related sleep disturbances are associated with biological changes, such as immunological dysregulation (2). In healthy individuals, data indicate a connection between sleep disturbances and enhanced systemic and cellular inflammation, including increased pro-inflammatory transcription in circulating leukocytes (2, 7, 8). In cancer patients, studies on the connection between immune activity and sleep disturbances are rare (2) and have produced heterogeneous results (9–11). For example, a study on women suffering from early-stage breast cancer who had recently finished primary treatment revealed that neither subjective sleep quality (SQ) nor sleep disturbances were significantly associated with serum levels of soluble tumor necrosis factor-α receptor II, interleukin-1 receptor antagonist, or C-reactive protein (10).

Heterogeneous findings in the research area of sleep–immune interactions in cancer may be related to the diversity of the studied cancer types and/or treatment-related characteristics (e.g., cancer stage) (12). However, inconsistent findings may also be attributable to the neglect of dynamic aspects related to the topic under study (13). Sleep–immune mechanisms are tightly bound to the circadian system that is composed of a network of transcriptional and/or posttranslational feedback loops (14, 15). This system, in turn, closely interwoven with cellular functions, affects both adaptive and innate immune function (16). Immunological processes, on the other hand, affect sleep factors *via* cytokines that act on neurons in sleep-involved brain regions (7, 17). Thus, the topic of sleep–immune interrelationships refers to a highly complex phenomenon. Approaching such complexity with inappropriate research designs and methods may result in heterogeneous findings (18).

To investigate the complex psychobiological aspects of sleep–immune interrelationships, e.g., temporal delays, response patterns, and directions of effects between variables, we suggest an idiographic and ecologically valid research approach based on qualitative methods and time-series analysis (18). In general, idiographic research approaches explore data at the individual level and thus overcome methodological problems related to intraindividual variability (e.g., day-to-day fluctuations) and interindividual heterogeneity (e.g., interindividual differences in stress-related cortisol responses) (19, 20). Indeed, when studying sleep–immune processes at the aggregating-nomothetic (group) level, these problems are inherent. Time-series analysis is a statistical tool that is often applied in idiographic research (19) and is capable of detecting temporal and directional relationships in complex processes (21). Approaches applying time-series analysis differ from conventional nomothetic approaches, e.g., by allowing the filtering of serial dependencies, which are prominent in circadian rhythms (21). Serial dependencies are a significant source of variance and may lead to false-positive or false-negative correlations when not statistically accounted for (21, 22).

There are various approaches in time-series analysis to assess and incorporate such autocorrelative effects within statistical models (22). The technique used in this study, centered moving average (CMA) smoothing, strips away random fluctuations and highlights the underlying trend in a time series (23). It can be applied to short time series (24).

Time-series analysis is part of our “integrative single-case design,” a study design that investigates stress system dynamics in a single individual under highly preserved everyday-life conditions (13, 18, 25, 26). In a previous article on the integrative single-case study presented here (same patient), we provided first evidence of real-life bidirectional relations between fatigue, mood, and urinary neopterin concentrations (27). Statistical analysis of 55 consecutive 12-h measurements (28 days) revealed that increases in urinary neopterin levels significantly preceded increases in fatigue intensity with a temporal delay of 60–72 h. Furthermore, increases in positive mood co-occurred with neopterin level increases and preceded decreases in neopterin concentrations with a delay of 132–144 h. Neopterin is released by macrophages upon interferon gamma (IFN-γ) activation and indicates cellular immune system activation and oxidative stress (28).

This article now focuses on the sleep-related time series and investigates the reciprocal relationship between subjective sleep and neopterin release. Subjective sleep estimates appear to be highly relevant to immunological changes (29, 30).

## Materials and Methods

### Study Design

This integrative single-case study is part of a larger project on the influence of emotionally meaningful incidents on stress system dynamics in breast cancer survivors. The applied study design is described in detail elsewhere (13, 18, 25, 26). Shortly before the study began, the patient was examined medically and psychologically to check for inclusion and exclusion criteria (27). During the study period of 28 days (from July 13th to August 9th, 2006), the patient collected her full urine output in 12-h intervals (from approximately 8:00 p.m. to 8:00 a.m. and from approximately 8:00 a.m. to 8:00 p.m.) to determine urinary neopterin and creatinine levels (total: 55 measurements). Moreover, the patient filled out various questionnaires at approximately 8:00 a.m. and again at approximately 8:00 p.m. to measure psycho-behavioral everyday-life variables such as emotional states, daily routine, and illness-related issues. The sleep questionnaire was answered by the patient every morning at approximately 8:00 a.m. and dealt with her sleep the preceding night. Once a week, the patient brought the frozen (−20°C) urine sample aliquots to the laboratory where they were stored at −70°C until analysis. During these clinical appointments, the general health status of the patient was monitored, and the patient was interviewed regarding psychosocial incidents of the previous week. For the purpose of this study, nighttime neopterin measurements and subjective sleep variables were used, each resulting in time series of 28 measurements.

The patient gave written informed consent to her participation and to the publication of data in accordance with the Declaration of Helsinki, and the Institutional Review Board of the Freiburg University approved the design.

### Measurement of Continuous Sleep Variables

Continuous day-to-day sleep assessment was based on subjective sleep ratings. Specifically, each morning at approximately 8:00 a.m., the patient answered questions about her prior night’s sleep. These questions were based on the Deutsche Gesellschaft für Schlafforschung und Schlafmedizin (DGSM) morning protocols (31) and correspond to items from Anglophone sleep diaries, such as the Karolinska Sleep Diary (32) and the Consensus Sleep Diary (33). The open response questions of this sleep questionnaire were as follows:

1. When did you turn out the light last night?
2. How long did it take you to fall asleep (min)?
3. How many times did you wake up during the night?
4. How long, in total, were you awake during the night?
5. How many hours did you sleep last night?
6. When did you wake up this morning?

The sleep questionnaire, furthermore, included two items answered on a 5-point-Likert-scale:

7. Compared with your average quality of sleep, how did you sleep last night?
8. How restful and refreshing was your night’s sleep?

From this sleep questionnaire, the time-series variables “sleep quality” (SQ), “sleep recreational value” (SRV), “total sleep time” (TST), “total wake time” (TWT), and “awakenings during sleep period” (ADS) were formulated.

### Measurement of Neopterin per Creatinine

The consecutive urinary neopterin and creatinine levels were determined using high-pressure liquid chromatography (HPLC) (Model ProStar 210 Solvent Delivery Modul; Varian Associates, Palo Alto, CA, USA), as previously described (34). All 55 12-h urinary aliquots were measured in one single run. For the purpose of this study, we only used those 28 urinary aliquots that corresponded to the 12-h intervals from approximately 8:00 p.m. to approximately 8:00 a.m. (nighttime samples). Urinary neopterin concentrations were expressed in micromolars per molar creatinine to compensate for variations in urine density. To check for possible measurement errors, urinary neopterin and creatinine concentrations were analyzed in five independent runs, using a new aliquot for each determination. These five independent urinary neopterin time series were averaged.

### Statistical Analyses

Time series (*n* = 28) were analyzed using IBM SPSS Statistics 23. To determine if a change in one variable significantly preceded a change in another variable, and to identify temporal delays and directions of effects between urinary neopterin and sleep variables, cross-correlation function (CCF) was performed at lag 0 and at higher lags up to ±7. CCF was considered statistically significant when the *p* < 0.05 criterion was met.

Beforehand, time series were stationarized through differencing and logarithmizing whenever necessary. Whenever autocorrelations were significant, time series were pre-whitened by CMA smoothing to deal with serial dependencies. An initial span of four values was specified for computing averages. To produce non-missing values at the endpoints of the original time series, a second span of two values was defined (35). CMA smoothing residuals were identified by subtracting the smoothed series from the original series (35, 36). These residuals were then used for CCF calculations (37).

## Results

Time-series data were based on 28 measurements (*n* = 28, no missing data). No signs of infection were observed during the study period. The patient under study took aspirin^®^ (acetylsalicylic acid, two tablets, 500 mg each) once during this study’s observation period, in night 14.

When averaged over the whole study period, the patient’s SQ had a mean value of 2.1 ± 1.5 (range: 0–4) and SRV showed a mean level of 1.2 ± 1 (range: 0–3). TST averaged out at 7.4 ± 1.6 (range: 5.1–11.2) hours per night. The mean value of TWT accounted for 21.1 ± 18.4 (range: 0–80) minutes per night. On average, the patient woke up 1.4 ± 0.8 (range: 0–3) times during the night (ADS). Urinary neopterin showed a mean concentration of 172.9 ± 25.7 (range: 131.6–226) μmol/mol creatinine (Table 1).

**Table 1:** Descriptive statistics of urinary neopterin concentrations and sleep variables (*n* = 28 consecutive measurements).

Parameter	Mean ± SD*	Range
Urinary neopterin (μmol/mol creatinine)	172.9 ± 25.7	131.6–226
Sleep quality	2.1 ± 1.5	0–4
Sleep recreational value	1.2 ± 1	0–3
Total sleep time (h)	7.4 ± 1.6	5.1–11.2
Total wake time (min)	21.1 ± 18.4	0–80
Awakenings during sleep period	1.4 ± 0.8	0–3

*SD: Standard deviation

**Figure 1:**
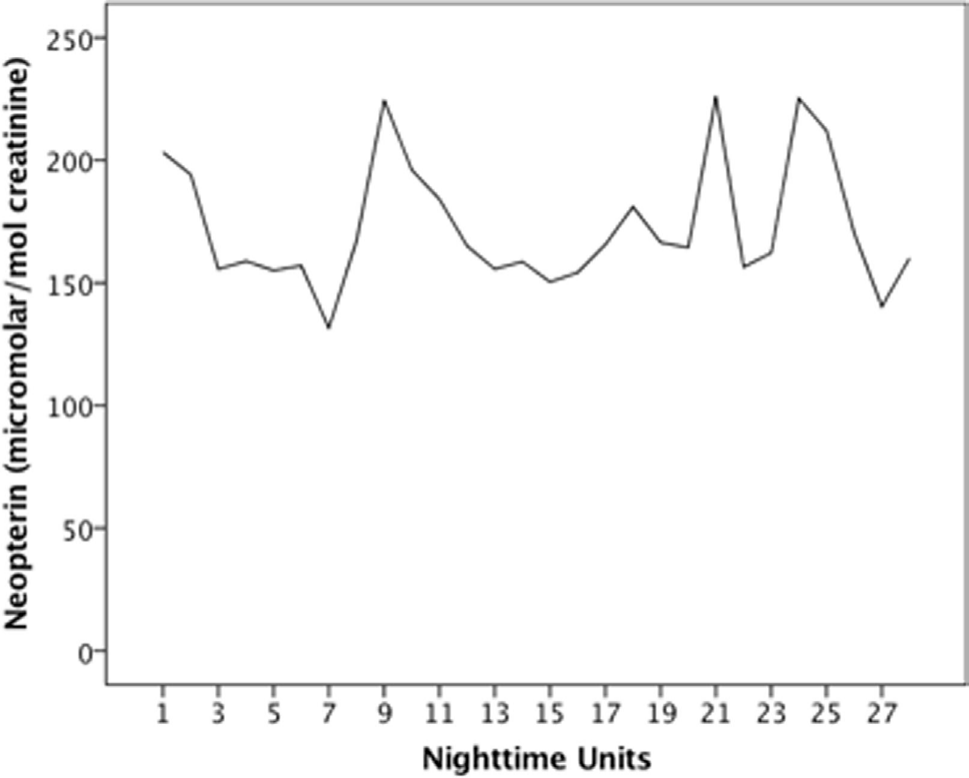
Time series of urinary neopterin. Time series covers night samples (8:00 p.m. to 8:00 a.m.) of a 28-day period during which the patient collected her full urine output in 12-h intervals.

**Figure 2:**
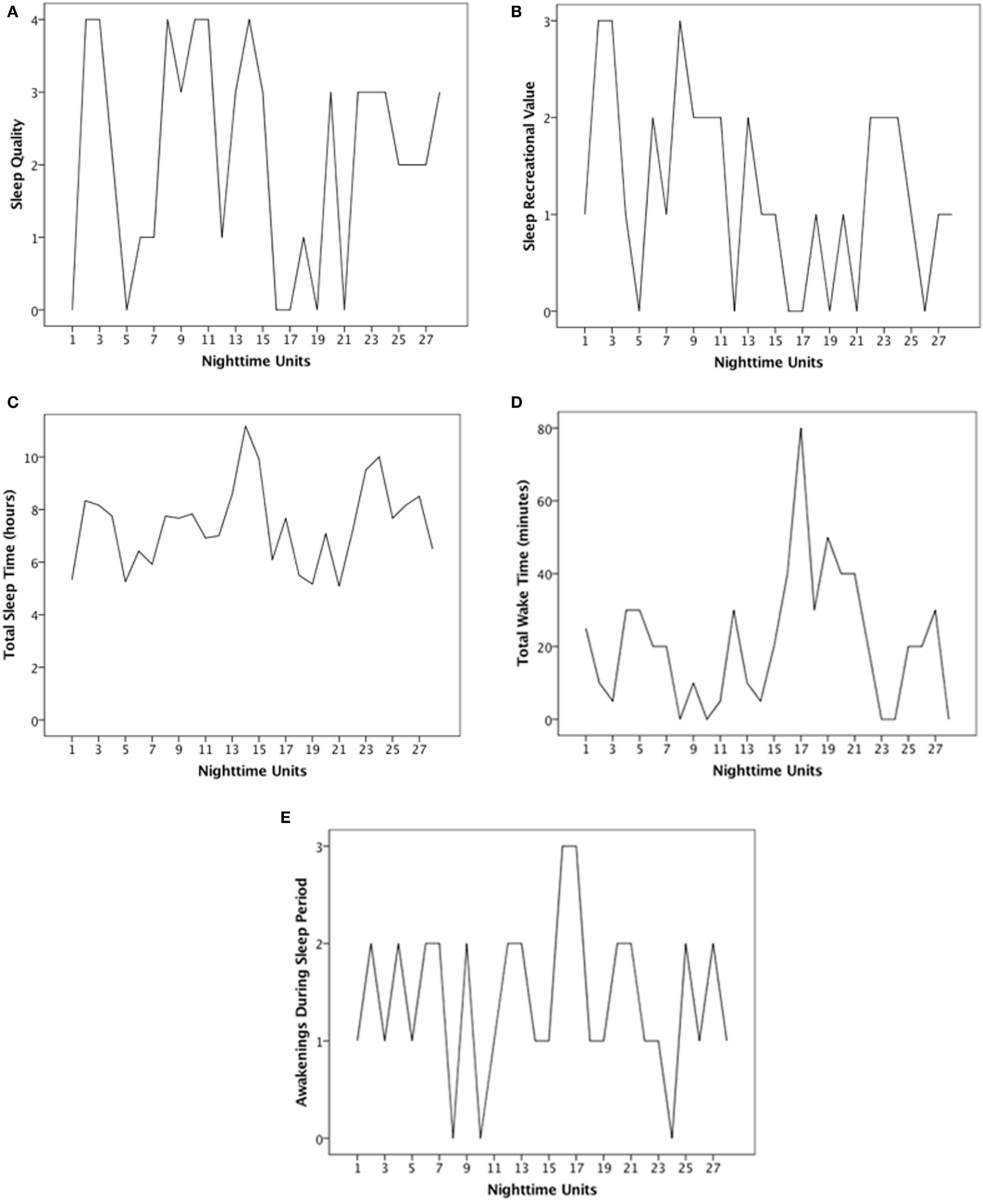
Time series of sleep variables. Time series display subjective ratings of **(A)** sleep quality (SQ), **(B)** sleep recreational value (SRV), **(C)** total sleep time (TST), **(D)** total wake time (TWT), and **(E)** awakenings during sleep period (ADS). Each time series covers a period of 28 days during which the patient answered questions about her prior night’s sleep each morning at approximately 8:00 a.m.

Figure 1 shows the time series of urinary neopterin per creatinine concentrations, which was stationary and needed no pre-whitening before cross-correlation with sleep variables. The raw time series of the five sleep variables under study are presented in Figures 2A–E. The time series of SQ, SRV, TST, and ADS were stationary. The time series of TWT was not stationary and thus had to be differenced. The time series of SQ, SRV, and TST showed no significant autocorrelations. Due to significant autocorrelation functions, TWT and ADS needed to be pre-whitened *via* CMA smoothing.

**Figure 3:**
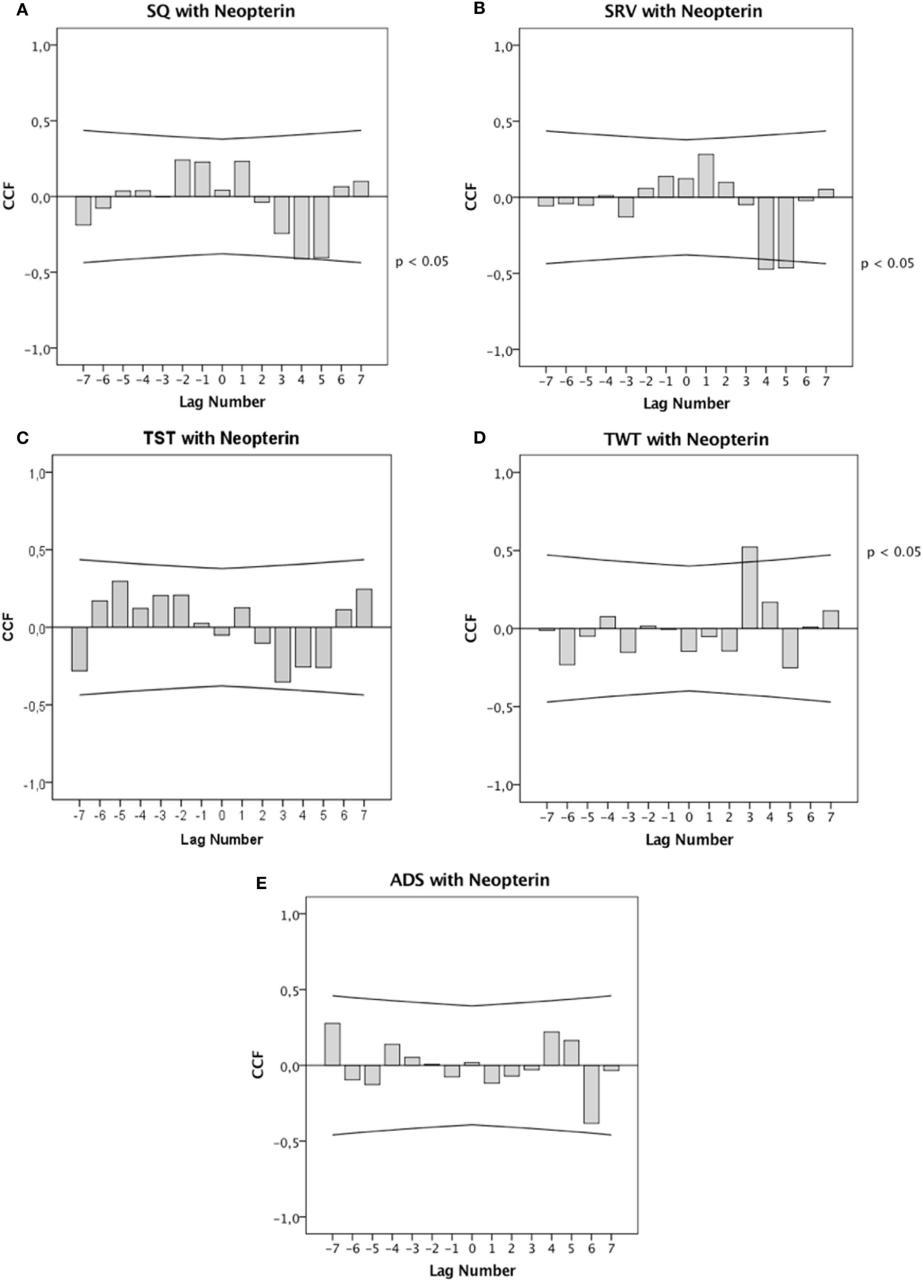
Cross-correlation functions (CCF). CCF between **(A)** sleep quality (SQ), **(B)** sleep recreational value (SRV), **(C)** total sleep time (TST), **(D)** total wake time (TWT), **(E)** awakenings during sleep period (ADS), and urinary neopterin levels. Each lag represents a time interval of 24 h. Cross-correlation coefficients (bars) reaching the upper or lower confidence limits (lines) are significant at *p* < 0.05.

The CCF in Figure 3A shows that increased SQ preceded significant decreases in urinary neopterin concentrations by 96–120 h (lag 4: *r* = −0.411; *p* = 0.044). Cross-correlational analysis further revealed that increased SRV significantly preceded decreases in urinary neopterin levels with temporal delays of 96–120 h (lag 4: *r* = −0.472; *p* = 0.021) and 120–144 h (lag 5: *r* = −0.464; *p* = 0.026) (see Figure 3B). There was no statistically significant connection between TST and urinary neopterin levels (see Figure 3C). As presented in Figure 3D, increases in TWT significantly preceded increases in urinary neopterin concentrations with a temporal delay of 72–96 h (lag 3: *r* = 0.522; *p* = 0.009). No statistically significant association between ADS and urinary neopterin levels was found (see Figure 3E).

## Discussion

This integrative single-case study on a breast cancer survivor suffering from CaRF and depression shows that subjective sleep factors preceded cellular immune activity changes with temporal delays far exceeding time intervals known from the current sleep–immune literature. More specifically, elevations in SQ and SRV were followed by decreases in urinary neopterin concentrations after 96–120 and 120–144 h. Furthermore, elevations in TWT preceded increases in urinary neopterin concentrations after 72–96 h. Taken together, this study shows that decreases in urinary neopterin concentration occur whenever the patient describes her sleep as qualitatively good, restful, and refreshing, whereas urinary neopterin concentration increases were related to longer wake times, a negative sleep factor.

Neopterin is a marker of enhanced cellular immune activation and oxidative stress that has been associated with poorer health outcomes and lower survival rates in disorders such as breast cancer (28, 38). We thus suggest that urinary neopterin concentration decreases in the patient under study are related to beneficial health consequences, whereas neopterin increases represent deteriorating health effects.

In line with this suggestion is the finding that these effects of positive and negative sleep factors on urinary neopterin levels are comparable to the stress effects identified in a prior study on a patient suffering from systemic lupus erythematosus (SLE) (25, 26), a chronic inflammatory disease (39). Specifically, in that patient with SLE, the occurrence of positive, emotionally fulfilling incidents preceded ultimate urinary neopterin concentration decreases after 84 h (26), whereas the occurrence of negative, emotionally painful incidents preceded ultimate increases in urinary neopterin levels 60 h later (25).

By applying time-series analysis on high-frequent measurements within a time frame of 28 days, this study reveals that significant sleep-to-immune changes occurred after 72–144 h, with the majority of significant changes evolving after 96–120 h. At first sight, these time intervals are quite long when compared with clearly shorter temporal delays reported in laboratory studies [e.g., 1–9 h in Ref. (40)]. However, laboratory studies, as a matter of principle, cannot reveal such long time intervals due to an artificial predetermination of restricted time frames in experimental settings. The design of the current exploratory study, by contrast, allows the sleep–immune interplay, which depends on strong external zeitgebers (14) (e.g., light, social interactions, and physical activity) and higher order psychophysiological processing, to unfold dynamically under realistic everyday-life conditions (41). We suggest that such complex sleep–immune aspects are responsible for the long time intervals identified in this study.

As with experimental designs, quasi-experimental aggregating-nomothetic research approaches also fall short of the complexity of the topic under study. Assessment of single blood samples (29), data collection procedures with poor intra- and/or interindividual time coordination (42) and disregard of serial dependencies all exemplify reductionist tendencies characteristic of common data-averaging approaches (21). Another critical issue related to such approaches is that they do not allow testing without *a priori* methodological restriction (e.g., pre–post design) whether immunological processes affect sleep factors, whether sleep factors affect immunological processes, and whether reciprocal relationships exist (21). Indeed, directions of effects need to be seen as key aspects within the sleep–immune framework (7, 14, 17). Time-series data, on the other hand, are especially well suited to investigate the sequencing of change across time (21). For the patient of this study, the question of the directionality of effects can be answered: sleep typically preceded changes in neopterin.

As to the limitations of this study, first, our data are restricted to subjective sleep ratings, which increase the risk of response bias due to retrospective recall (43). In this regard, objective sleep assessment (e.g., actigraphy) would have revealed a more complete picture. A second limitation of the current investigation is the shortness of time-series data (*n* = 28), which prevented us from using more powerful modeling approaches such as autoregressive integrated moving average (ARIMA) modeling, where a minimum of 50 measurements is recommended for accurate estimation (44). Third, the findings may not be generalized yet due to the study’s exploratory character and the *n* = 1 design.

However, case numbers and sample size are not everything. Ecological validity is another important factor when findings from studies on human beings are to be generalized (45). The higher a study’s ecological validity, the higher its potential to generalize findings across settings and conditions (45). Our study, which was developed to achieve conditions as close to the subject’s natural environment as possible, displays particularly high ecological validity (18). In other words: observing “life as it is lived”—naturally, outside of the laboratory setting—is an essential strength of our study design.

## Concluding Remarks

This study highlights the advantages of idiographic research approaches, which take a more “microscopic” perspective when studying the complex connections between sleep and immune processes under natural living conditions.

## Ethics Statement

This study was carried out in accordance with the recommendations of the Institutional Review Board of the Freiburg University with written informed consent from the subject. The subject gave written informed consent in accordance with the Declaration of Helsinki. The protocol was approved by the Institutional Review Board of the Freiburg University.

## Author Contributions

Substantial contributions to the conception or design of the work, the acquisition, analysis, or interpretation of data for the work; final approval of the version to be published; and agreement to be accountable for all aspects of the work in ensuring that questions related to the accuracy or integrity of any part of the work are appropriately investigated and resolved: MS, CB, KF, SP, HB, FO-P, DF, and CS. Drafting the work or revising it critically for important intellectual content: MS, SP, HB, FO-P, DF, and CS.

## Conflict of Interest Statement

The authors declare that the research was conducted in the absence of any commercial or financial relationships that could be construed as a potential conflict of interest.

## Abbreviations

ADS, awakenings during sleep period; ARIMA, autoregressive integrated moving average; CaRF, cancer-related fatigue; CCF, cross-correlation function; CES-D, Center for Epidemiologic Studies Depression Scale; CMA, centered moving average; CRP, C-reactive protein; DGSM, Deutsche Gesellschaft für Schlafforschung und Schlafmedizin; HPLC, high pressure liquid chromatography; IFN-γ, interferon gamma; IL-1RA, interleukin-1 receptor antagonist; SLE, systemic lupus erythematosus; SQ, sleep quality; SRV, sleep recreational value; sTNF-R, soluble tumor necrosis factor-α receptor; TST, total sleep time; TWT, total wake time.
